# Religious Involvement and Marijuana Use for Medical and Recreational Purposes

**DOI:** 10.1177/0022042618770393

**Published:** 2018-04-21

**Authors:** Amy M. Burdette, Noah S. Webb, Terrence D. Hill, Stacy Hoskins Haynes, Jason A. Ford

**Affiliations:** 1 Florida State University, Tallahassee, USA; 2 University of Arizona, Tucson, USA; 3 Mississippi State University, Mississippi State, USA; 4 University of Central Florida, Orlando, USA

**Keywords:** United States, religion, religious service attendance, substance use, medical marijuana, poor health

## Abstract

In this article, we use data from the 2016 National Survey on Drug Use and Health (NSDUH) to examine the association between religious involvement and marijuana use for medical and recreational purposes in U.S. adults (*N* = 41,517). We also consider whether the association between religious involvement and marijuana use varies according to personal health status. Our results show that adults who attend religious services more frequently and hold more salient religious beliefs tend to exhibit lower rates of medical and recreational marijuana use. We also find that these “protective effects” are less pronounced for adults in poor health. Although our findings confirm previous studies of recreational marijuana use, we are the first to examine the association between religious involvement and medical marijuana use. Our moderation analyses suggest that the morality and social control functions of religious involvement may be offset under the conditions of poor health.

## Introduction

Marijuana use for medical and recreational purposes is on the rise (Carliner et al., 2017; Caulkins, Kilmer, Reuter, & Midgette, 2015; Fairman, 2016; Han et al., 2017; Hasin et al., 2015). Comprehensive public medical marijuana and cannabis programs are now available in 29 states, the District of Columbia, Guam, and Puerto Rico. It should come as no surprise that public attitudes have also liberalized over time, with strong majorities of U.S. adults supporting medical marijuana use and the legalization of marijuana (Gallup, 2017; Quinnipiac University Poll, 2017). Despite these trends, prescribing marijuana remains illegal under federal law where it is categorized as a Schedule I substance under the Controlled Substances Act of 1970. In the eyes of the federal government, marijuana use has no medical value and is likely to lead to abuse and addiction (Chaudhry, Hengerer, & Snyder, 2016). While the American Medical Association’s House of Delegates (2016) has urged the federal government to review marijuana’s status as a Schedule I substance to facilitate research on the potential therapeutic value of marijuana, most medical organizations also hold that cannabis is a dangerous public health concern.

In the context of changing attitudes and behavior and conflicting state and federal statutes, we must prioritize studies that focus on the social patterning of marijuana use for medical and recreational purposes. Along these lines, we emphasize the role of religious involvement. Although numerous studies show that religious involvement is associated with lower rates of substance use and abuse (Chitwood, Weiss, & Leukefeld, 2008; Hill, Burdette, Weiss, & Chitwood, 2009; Koenig, King, & Carson, 2012; Krause, Pargament, Ironson, & Hill, 2017), a surprisingly small proportion of this research base has been devoted to general marijuana use among U.S. adults. We were also unable to find any studies of religious involvement and medical marijuana use. Nevertheless, religious involvement (e.g., religious attendance and religious beliefs) has long been associated with lower rates of recreational or illicit marijuana use (Bartkowski & Xu, 2007; Hill et al., 2009; Koenig et al., 2012; Longest & Vaisey, 2008; Nonnemaker, McNeely, & Blum, 2003), especially among adolescents and young adults.

Previous research has identified several compelling theories for why religious involvement might be associated with lower rates of substance use. The *socialization perspective* suggests that involvement in religious institutions exposes adherents to specific moral directives and general religious doctrines that are supported by the authority of religious traditions and sacred texts. Ongoing exposure to these tenets may lead individuals to internalize specific religious messages that discourage substance use and abuse (Adamczyk & Palmer, 2008; Bartkowski & Xu, 2007; Chitwood et al., 2008; Ford & Hill, 2012; Hadaway, Elifson, & Petersen, 1984; Hill et al., 2009; Longest & Vaisey, 2008). Many religious groups also adhere to general religious ideologies that sanctify the body and promote the importance of physical health as a means of religious commitment. For example, religious groups draw on scripture suggesting that the “body is the temple of the Holy Spirit” to warn against a variety of health-relevant behaviors, including alcohol consumption, tobacco smoking, illicit drug use, and risky sexual behaviors (Ford & Hill, 2012; Hill et al., 2009).

The *authority perspective* suggests that religious involvement may also deter substance use by encouraging a general deference to authority, conformity to societal norms, and adherence to laws. Numerous biblical passages counsel adherents to submit to various “authorities” and “ordinances” (e.g., Hebrews 13:17; Peter 2:13-14; Romans 13:1-7). For instance, Romans (13:1-2) advises:

Let every soul be subject to the governing authorities. For there is no authority except from God, and the authorities that exist are appointed by God. Therefore whoever resists the authority resists the ordinance of God, and those who resist will bring judgment on themselves.

Those who are active within religious institutions may favor conformity through fear of divine retribution, internalized moral codes, guilt avoidance, and the social context of obedient peer networks (Welch, Tittle, & Grasmick, 2006). If religious individuals are more deferential to authority than others, they may be more likely to obey laws prohibiting illicit substance use and the use of prescription drugs in the medically prescribed manner.

The *control perspective* suggests that religious involvement may also reduce the risk of substance use through processes related to social control and social support. Frequent religious attendance creates opportunities for regular contact with adherents, which could imply the potential for behavioral monitoring, detection of counter-normative behavior, and possible social sanctions (Sherkat & Wilson, 1995). Religious involvement is associated with direct and indirect exposure to social sanctions (e.g., gossip, ostracism, and formal punishments) that function to elevate the costs (actual and perceived) associated with substance use, which presumably limit access and use. Furthermore, religious involvement may reduce substance use by embedding individuals in reference groups that tend to espouse anti-substance use norms and exhibit low levels of substance use and high rates of abstinence (Hill et al., 2009). Religious involvement may also lead to lower levels of substance use through supportive relationships with coreligionists (Carrico, Gifford, & Moos, 2007; Humphreys & Gifford, 2006). Studies show that religious involvement is associated with larger and more diverse social networks, more contact with network members, more extensive family ties, and more types of social support (Ellison & George, 1994; Rote, Hill, & Ellison, 2013). Larger social networks, especially those consisting of coreligionists, may discourage marijuana use through the provision of informational, emotional, and instrumental support.

Finally, the *self-regulation perspective* suggests that religious involvement may be associated with lower levels of substance use by fostering self-control and generic self-regulatory capacities (DeWall et al., 2014; McCullough & Willoughby, 2009; Nasim, Utsey, Corona, & Belgrave, 2006; Pascoe, Hill, Mossakowski, & Johnson, 2016; Smith, 2003). In a systematic review of empirical research, McCullough and Willoughby (2009) show that religious individuals consistently score higher than their less religious counterparts on measures of self-control (e.g., ability to control one’s impulses, appetites, and emotions). They demonstrate that self-control appears to be one of the mechanisms through which religious involvement is associated with substance use among adolescents. Drawing on a diverse set of samples, including college students and community-dwelling adults in the United States and Asia, DeWall and colleagues (2014) find that self-control mediates the association between religious involvement and a variety of substance use behaviors.

Although religious involvement is likely to be associated with lower rates of marijuana use, this association could be contingent on health status. Even in the context of contentious political debates, marijuana use has been increasingly medicalized and is now viewed as an acceptable treatment for a range of health conditions in several states (Wilkinson & D’Souza, 2014). For this reason, marijuana use is often more common among adults reporting fair or poor health (Compton, Han, Hughes, Jones, & Blanco, 2017; Ford, 2014; Lin, Ilgen, Jannausch, & Bohnert, 2016). On one hand, religious prohibitions against substance use, the precarious legality of medical marijuana, and alternative forms of coping may lead religious adherents in poor health to dismiss marijuana as a viable form of medical treatment. Limited research suggests that religious individuals are more opposed to complementary and alternative medicine in comparison with those individuals who identify as spiritual only (Ellison, Bradshaw, & Roberts, 2012). In addition, Evans (2006) suggests that religious individuals may disapprove of certain medical technologies in part because they see potential value in suffering, as compared with nonreligious individuals, which tend to view suffering as something to stop as quickly as possible. These findings suggest that religious individuals in poor health may be more likely to rely on traditional medical treatments during times of illness and less likely to use marijuana medically.

On the other hand, the realities of coping with a chronic illness may reduce the influence of religious prohibitions as individuals look for effective ways to deal with pain and suffering, particularly when marijuana use is recommended by a medical professional. There is some broader theoretical speculation that secularization (the decline of religious institutions) has contributed to medicalization (the social dominance of medicine; Bull, 1990; Conrad, 1992; Turner, 1984). The idea is that “medicine has ‘nudged aside’ or ‘replaced’ religion as the dominant moral ideology and social control institution in modern societies” (Conrad, 1992, p. 213). When religious people are in poor health, the moral authority and social control functions of religious institutions may be challenged by the “moral domination” of medical institutions. If religion says no, and medicine says yes, which institution is likely to direct the marijuana use of religious people under the conditions of poor health? The secularization/medicalization perspective suggests that the “protective effects” of religious involvement will be attenuated by poor health.

This hypothesis is bolstered by research suggesting that highly religious individuals are more likely to trust physicians (Benjamins, 2006) and less likely to question authority (Welch et al., 2006). The corollary is that religious involvement will matter more for the marijuana use of healthy people because poor health is a common precondition for treatment within medical institutions and, by extension, increased exposure to potentially conflicting values.

In this article, we use data collected from a large national sample of U.S. adults to examine the effects of religious involvement on marijuana use for medical and recreational purposes. We contribute to previous research in several ways. First, our analyses focus on adults, not adolescents or young adults. Second, we examine associations with medical marijuana, which has been unexplored in previous studies. Finally, we formally test whether the association between religious involvement and marijuana use varies as a function of health status. Based on previous theory and research, we expect to find that greater religious involvement will be associated with lower rates of marijuana use for medical and recreational purposes (Hypothesis 1[H1]). We also test two competing hypotheses concerning the moderating influence of health status. One hypothesis suggests that the institution of religion will trump the institution of medicine: The association between religious involvement and medical marijuana will tend to be more pronounced for adults in poor health (Hypothesis 2 [H2]). The other suggests that the institution of medicine will trump the institution of religion: The association between religious involvement and medical marijuana will tend to be less pronounced for adults in poor health (Hypothesis 3 [H3]).

## Method

### Data

This study uses data from the 2016 National Survey on Drug Use and Health (NSDUH), which is an annual, cross-sectional survey (Center for Behavioral Health Statistics and Quality, 2017a). The NSDUH utilizes a multistage, state-based probability sampling design to collect data concerning substance use and associated health conditions in a representative sample of adolescent and adult members of the U.S. civilian, noninstitutionalized population.

Each year, the NSDUH surveys approximately 70,000 individuals aged 12 years and older, allocating the sample size relatively equally across multiple age groups: individuals aged 12 to 17, 18 to 25, 26 to 34, 35 to 49, and 50 or older. The overall response rate for the 2016 survey was 68.44%. Because children and adolescents make up less than 1% of registered medical marijuana participants (Fairman, 2016), the current study is limited to adults aged 18 years and older, which yields a sample size of 42,625. Three focal variables were subject to missing data: religious service attendance (0.90%), religious salience (2.34%), and self-rated health (0.01%). After losing 1,078 respondents (2.60%) through listwise deletion, our final analytic sample was reduced to 41,517 adults. Additional information about the NSDUH sampling methods and survey techniques can be found elsewhere (Center for Behavioral Health Statistics and Quality, 2017b).

### Measures

The dependent variable of interest is a three-outcome nominal variable that assesses the prevalence of past-year marijuana use. Respondents are classified as *nonusers* (those who did not use marijuana in the previous 12 months), *recreational users* (those who used marijuana in the previous 12 months, but did so without a doctor’s recommendation), and *medical users* (those who used marijuana in the previous 12 months with a doctor’s recommendation).

The main predictor variables of interest are religious service attendance and religious salience. Religious service attendance is measured as a continuous variable based on the responses to the following question: “During the past 12 months, how many times did you attend religious services? Please do not include special occasions such as weddings, funerals, or other special events in your answer.” Possible responses to this question included 1 = *0 times*, 2 = *1 to 2 times*, 3 = *3 to 5 times*, 4 = *6 to 24 times*, 5 = *25 to 52 times*, and 6 = *more than 52 times*. To provide more illustrative categories, we have renamed the above responses: “never” = *0 times*, “few times a year” = *1 to 2 times*, “< once a month” = *3-5 times*, “once a month+” = *6-24 times*, “weekly” = *25-52 times*, and “weekly+” = *more than 52 times*.

Religious salience is measured as a two-item index (Cronbach’s α = .895), in which higher scores indicate higher levels of religious salience. The two items assess the role that religious beliefs may play in one’s life, with responses ranging from 1 = *strongly disagree* to 4 = *strongly agree*. Respondents were asked to indicate their level of agreement with the following two statements: (a) “Your religious beliefs are a very important part of your life” and (b) “Your religious beliefs influence how you make decisions in your life.”

Previous research identifies a number of correlates of religious service attendance, religious salience, and marijuana use (Bartkowski & Xu, 2007; Longest & Vaisey, 2008; Nonnemaker et al., 2003). To account for such factors, our study controls for the following variables: *gender* (1 = male, 0 = female), *race/ethnicity* (0 = non-Hispanic White, 1 = non-Hispanic Black, 1 = Hispanic, 1 = Asian, 1 = Other Race), *age* (1 = 18-29 years, 1 = 30-49 years, 0 = 50+ years), *level of education* (1 = less than high school, 1 = high school, 1 = some college, 0 = college), *marital status* (0 = married, 1 = widowed, 1 = divorced or separated, 1 = never married), *employment status* (0 = employed full-time, 1 = employed part-time, 1 = unemployed, 1 = not in the labor force), and *urbanicity* (0 = urban, 1 = suburban, 1 = rural). We also control for *self-rated health*, which is measured with the following question: “Would you say your health in general is excellent, very good, good, fair, or poor?” This item was recoded into two groups: *good health* (0 = excellent, very good, or good health) and *poor health* (1 = fair or poor health). Consistent with similar work (e.g., Compton et al., 2017), we include a measure for *state law*, which assesses whether or not the respondent was residing in a state that allowed the use of medical marijuana (1 = resides in state with approved medical use, 0 = does not reside in state with approved medical use).

### Statistical Procedures

**Figure 1.**
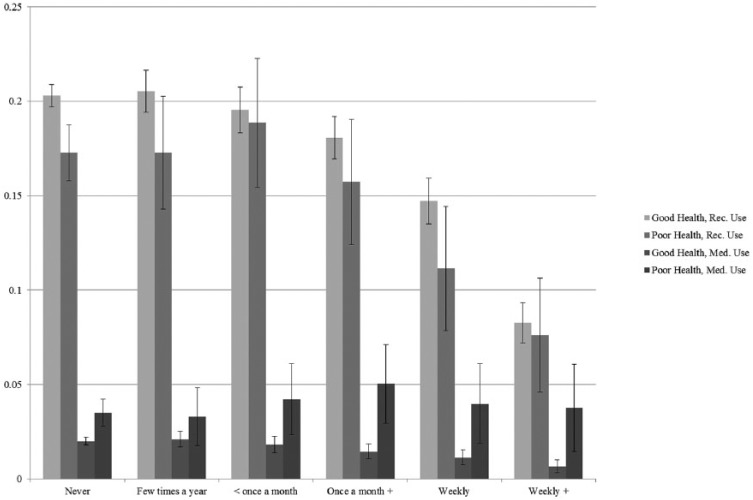
Adjusted probabilities of marijuana use by self-rated health and religious attendance.

**Figure 2.**
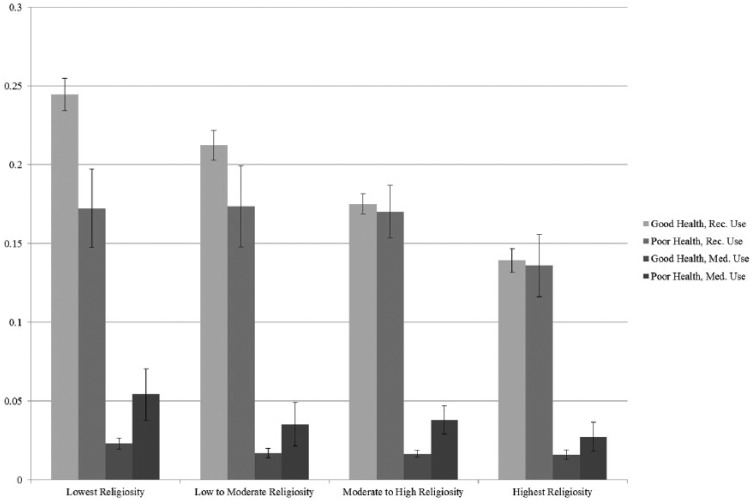
Adjusted probabilities of marijuana use by self-rated health and religious salience.

Our focal analyses are presented in two tables and two figures. Table 1 provides descriptive statistics for all study variables, including variable ranges, sample means or percentages, and standard deviations. Table 1 also displays our study variables stratified by type of marijuana use. In subsequent multivariate analyses (Table 2), we employ multinomial logistic regression to model type of marijuana use as a function of religious involvement. Model 1 assesses religious variations in recreational marijuana use as compared with no marijuana use in the past 12 months, controlling for sociodemographic characteristics. Models 2 and 3 examine whether the impact of religious involvement on recreational marijuana use varies by health status. Model 4 assesses religious variations in medical marijuana use as compared with no marijuana use. Models 5 and 6 examine whether the impact of religious involvement on medical marijuana use varies by health status. Finally, Figures 1 and 2 present adjusted probabilities for each of our outcomes as a function of our two measures of religious involvement and health status. The probabilities and confidence intervals (CIs) presented in our figures were generated using the margins command in Stata (StataCorp, 2015) and are adjusted for the sociodemographic controls presented in Table 1. In these figures, point estimates for each category of religious involvement are presented graphically, with vertical lines depicting 95% CIs around each probability estimate.

**Table 1.** Descriptive Statistics by Type of Marijuana Use.

	Full sample		Nonusers		Recreational users		Medical users	
	(*N* = 41,517)		(*n* = 33,090)		(*n* = 7,605)		(*n* = 822)	
	*M* (%)	*SD*	*M* (%)	*SD*	*M* (%)	*SD*	*M* (%)	*SD*
Type of use								
None	79.70							
Recreational	18.32							
Medical	1.98							
Focal variables								
Religious attendance	2.69	1.83	2.86	1.87	2.05	1.45	2.00	1.44
Religious salience	2.76	1.03	2.87	1.01	2.35	1.01	2.38	1.04
Control variables								
Male	46.61		44.51		54.53		57.91	
Female	53.39		55.49		45.47		42.09	
White	61.30		61.25		61.79		59.12	
Black	12.80		12.30		15.00		12.65	
Hispanic	16.37		17.01		13.62		16.18	
Asian	4.31		4.83		2.29		2.19	
Other race	5.21		4.62		7.30		9.85	
18-29	41.29		35.43		65.72		51.22	
30-49	37.89		40.26		27.71		36.37	
50+	20.82		24.31		6.57		12.41	
Less than high school	12.55		12.66		12.19		11.31	
High school	26.43		26.07		27.71		28.95	
Some college	34.02		32.71		38.79		42.70	
College graduate	27.01		28.56		21.31		17.03	
Married	41.06		46.52		19.32		22.38	
Widowed	3.02		3.59		0.70		1.70	
Divorced or separated	11.12		11.59		8.61		15.69	
Never married	44.80		38.30		71.37		60.22	
Employed full-time	51.98		52.32		51.37		43.92	
Employed part-time	15.84		14.75		20.51		16.30	
Unemployed	6.07		5.22		9.32		10.58	
Not in labor force	26.11		27.71		18.79		29.20	
Urban	42.35		41.76		44.33		47.81	
Suburban	50.06		50.36		49.19		45.86	
Rural	7.59		7.88		6.48		6.33	
Good health	88.85		88.79		90.14		79.08	
Poor health	11.15		11.21		9.86		20.92	
State law	50.66		48.71		56.25		77.25	

**Table 2.** Multinomial Logistic Regression Predicting Type of Marijuana Use.

	Recreational users versus nonusers			Medical users versus nonusers		
	Model 1	Model 2	Model 3	Model 4	Model 5	Model 6
Focal variables						
Religious attendance	0.87***	0.86***	0.87***	0.87***	0.83***	0.87***
Religious salience	0.77***	0.77***	0.75***	0.80***	0.80***	0.79***
Control variables						
Male	1.37***	1.37***	1.37***	1.72***	1.73***	1.72***
Black	1.12**	1.12**	1.12**	1.01	1.00	1.01
Hispanic	0.61***	0.61***	0.61***	0.70**	0.69**	0.70**
Asian	0.36***	0.36***	0.36***	0.33***	0.33***	0.33***
Other race	1.26***	1.26***	1.26***	1.60***	1.60	1.60***
18-29	3.61***	3.62***	3.64***	2.44***	2.46***	2.45***
30-49	2.02***	2.02***	2.03***	2.00***	2.00***	2.00***
Less than high school	0.95	0.95	0.95	0.94	0.93	0.94
High school	0.98	0.98	0.98	1.25	1.24	1.25
Some college	1.16***	1.16***	1.16***	1.69***	1.68***	1.69***
Widowed	0.88	0.87	0.87	1.34	1.32	1.34
Divorced or separated	1.81***	1.81***	1.81***	2.51***	2.50***	2.51***
Never married	2.34***	2.34***	2.34***	1.97***	1.97***	1.97***
Employed part-time	1.17***	1.17***	1.17***	1.19	1.19	1.19
Unemployed	1.24***	1.24***	1.24***	1.76***	1.76***	1.76***
Not in labor force	0.83***	0.83***	0.83***	1.38**	1.38**	1.38**
Suburban	0.91**	0.91**	0.91**	0.84*	0.84*	0.84*
Rural	0.80***	0.80**	0.80***	0.73*	0.73*	0.73*
Poor health	1.17**	1.01	0.71**	2.27***	1.44*	1.77*
State law	1.28***	1.28***	1.28***	3.45***	3.43***	3.45***
Interaction terms						
Religious Attendance × Poor Health		1.07*			1.24***	
Religious Salience × Poor Health			1.22***			1.10

*Note. N* = 41,517. Shown are odds ratios.

* *p* < .05. **p < .01. ***p < .001.

## Results

Table 1 presents descriptive statistics for all measures stratified by type of marijuana use. Overall, nearly 80% of respondents reported no marijuana use within the previous 12 months. Approximately 18% reported past-year recreational use, while roughly 2% reported past-year medical use. The average respondent reported attending religious services more than a few times a year, but less than once a month, and “agreed” that religious beliefs were an important part of his or her daily life and influenced his or her decision making. Both levels of religious service attendance and religious salience were notably lower among recreational and medical marijuana users. While recreational marijuana users reported self-rated health similar to that of the full sample, a much higher percentage of medical marijuana users reported being in poor health (21%).

With regard to other characteristics, the sample consists of non-Hispanic Whites (61%), non-Hispanic Blacks (13%), Hispanics (16%), Asians (4%), and other races/ethnicities (5%). Most respondents are female (53%), employed full-time (52%), report being in good health (89%), and reside in states that approve the medical use of marijuana (51%). Nearly half of the respondents are between the ages of 18 and 29 years (42%), have never been married (45%), and live in urban settings (42%), while roughly a third of the sample have completed some college (34%).

Multinomial logistic regression analyses are displayed in Table 2. Models 1 to 3 assess the odds of being a recreational marijuana user versus a nonuser. The odds of being a recreational user (as compared with a nonuser) are 13% lower for each unit increase in religious service attendance (odds ratio [OR] = 0.87; CI = [0.82, 0.91]; *p* < .001) and 23% lower for each unit increase in religious salience (OR = 0.77; CI = [0.74, 0.79]; *p* < .001), net of relevant demographic characteristics (Model 1). In Models 2 and 3, our interaction terms reach significance, indicating that the impact of both religious service attendance (OR = 1.07; CI = [1.01, 1.13]; *p* < .05) and religious salience (OR = 1.22; CI = [1.12, 1.33]; *p* < .001) on recreational marijuana use varies as a function of health status.^1^ Models 4 to 6 assess the odds of being a medical marijuana user versus a nonuser. Similar to recreational users, the odds of being a medical user (as compared with a nonuser) are 13% lower for each unit increase in religious service attendance (OR = 0.87; CI = [0.82, 0.91]; *p* < .001) and 20% lower for each unit increase in religious salience (OR = 0.80; CI = [0.74, 0.86]; *p* < .001), net of controls for background factors (Model 4). In Models 5 and 6, one of our interaction terms reaches significance, indicating that the impact of religious service attendance (OR = 1.24; CI = [1.12, 1.38]; *p* < .001) on medical marijuana use varies by health status.

We further explore the significant interactions between our measures of religious involvement and health status displayed in Models 2 and 3 and Models 5 and 6 of Table 2. We present adjusted probabilities for each of our outcomes of interest by frequency of religious service attendance and health status (Figure 1) and by religious salience and health status (Figure 2), accounting for the sociodemographic characteristics. In Figure 1, among respondents who report being in good health, the probability of engaging in medical and recreational marijuana use is significantly lower among those who attend services at least once a week as compared with those who attend religious services less frequently. Among respondents in poor health, the association between religious service attendance and marijuana use is less pronounced. Respondents who attend religious services more than once a week are less likely to use marijuana recreationally as compared with those who attend less than weekly. Similarly, those who attend religious services weekly are less likely to be recreational users as compared with those who attend less than once a month. Religious service attendance appears to have no impact on the probability of using medical marijuana among those in poor health. In Figure 2, among those respondents in good health, there is an inverse association between religious salience and the probability of recreational marijuana use. Conversely, religious salience appears to have no impact on the probability of using marijuana recreationally among those in poor health.

## Discussion

Although the inverse association between religious involvement and substance use in adolescence and young adulthood is well established (Chitwood et al., 2008; Hill et al., 2009; Koenig, McCullough, & Larson, 2001), few studies have focused on marijuana use in adulthood. The dearth of research centering on medical marijuana is particularly troublesome given rapidly changing attitudes and continued scientific debate (Rubens, 2014; Whiting et al., 2015). It is also unclear how the “protective effects” of religious involvement might interact with health status, one of the strongest predictors of medical marijuana use (Compton et al., 2017; Lin et al., 2016; Ryan-Ibarra, Induni, & Ewing, 2015).

Consistent with our first hypothesis, religious involvement is associated with reduced recreational and medical marijuana use. Our findings suggest that multiple measures of religiosity are important predictors of substance use. While frequency of religious attendance may indicate exposure to moral messages regarding substance use, religious salience may indicate the degree to which these messages have been internalized (Rohrbaugh & Jessor, 1975). Although our findings for recreational marijuana use are somewhat unsurprising given previous research on religious involvement and illicit substance use, our findings for medical marijuana use are remarkable. It may be that long-held religious prohibitions against substance use coupled with the unlawfulness of marijuana use at the federal level lead highly religious individuals to overlook marijuana as a practical alternative to traditional medical treatments.

Our results suggest that the impact of religious involvement on the likelihood of medical marijuana use is attenuated under the conditions of poor health. While these results are inconsistent with our second hypothesis, they lend support to our third hypothesis, which proposes that the moral authority and social control functions of religious institutions may be challenged by the “moral domination” of medical institutions. In this case, religious involvement is less effective in deterring marijuana use among adults in poor health. Somewhat surprisingly, our findings also show that the impact of religious involvement on the likelihood of *recreational* marijuana use is attenuated under the conditions of poor health. This finding illustrates the need for additional research to distinguish the *secularization/medicalization perspective* from other viable explanations. This finding may indicate that the influence of medicalization on the use of marijuana as medicine is so pervasive that it undermines the influence of religion during times of poor health, regardless if it is recommended by a doctor. Alternatively, the need for a means to cope with poor health could simply override the social costs associated with consuming marijuana.

We acknowledge that our study is limited in at least four key respects. First, although our measures of marijuana use are consistent with previous scholarship in this area (Cerdá, Wall, Keyes, Galea, & Hasin, 2012; Harper, Strumpf, & Kaufman, 2012; Hasin et al., 2015; Lin et al., 2016), they are simplistic. Future research in this area should consider more comprehensive measures. Second, it is also important to consider the influence of omitted variables on our key results. For example, Hill, Burdette, and Idler (2011) note that if individuals with certain conventional and risk-averse personality types are attracted to or selected into religious activities, personality selection processes could account for at least some of the effects of religious attendance. The NSDUH data do not allow for direct examination of these issues. Similarly, the NSDUH data do not include a measure of religious affiliation. While not accounting for religious affiliation may lead us to overestimate the influence of religious involvement, we must note that previous research has shown protective effects of religious involvement on recreational marijuana use when religious affiliation is held constant (e.g., Bartkowski & Xu, 2007; Burdette, Hill, Webb, Ford, & Haynes, 2018; Longest & Vaisey, 2008). Third, our data are based on self-reports, and respondents may be more likely to avoid reporting socially undesirable behaviors like substance use. However, NSDUH employs computer-assisted self-administered interview methods, which have been shown in previous studies to reduce underreporting of substance use (Harrison, Martin, Enev, & Harrington, 2007). Finally, because these data are cross-sectional, we cannot infer causality. Nevertheless, recent longitudinal research has found that while prior religious attendance is associated with lower rates of subsequent substance use, prior substance use is unrelated to subsequent religious attendance (Burdette et al., 2018).

Despite these limitations, our study contributes to previous research by examining the intersections of religious involvement, health status, and marijuana use in a nationally representative sample of U.S. adults. Our results suggest that religious involvement is associated with lower rates of recreational and medical marijuana use, especially among adults in good health. In light of these findings, additional empirical work is needed to understand the specific mechanisms linking religious involvement with reduced recreational and medical marijuana use. Research along these lines will provide a more complete understanding of the precise role of religious involvement in the context of increasing marijuana use.

## Notes

1. Some readers may be concerned that our analyses do not include an alpha correction. Because the strategy of adjusting for multiple tests is not universally accepted (see Nakagawa, 2004; Perneger, 1998; Rothman, 1990) and significantly reduces power and inflates the probability of Type II errors (i.e., accepting the null when it is false; Nakagawa, 2004; Perneger, 1998; Rothman, 1990), we have opted not to include an alpha correction in our focal analyses. Ancillary analyses applying the Bonferroni correction show that the interaction term presented in Model 2 is no longer significant when a more conservative statistical threshold is applied. All other focal variables remain statistically significant despite this correction. As a result, readers should interpret our results in Model 2 with caution and future research should continue to examine the interaction between religion and health status in relation to marijuana use.
